# A cross-cultural comparison of work engagement in the relationships between trust climate – Job performance and turnover intention: Focusing China and Pakistan

**DOI:** 10.1016/j.heliyon.2023.e19534

**Published:** 2023-08-30

**Authors:** Aini Aman, Muhammad Rafiq, Omkar Dastane

**Affiliations:** a Faculty of Economics and Management, Universiti Kebangsaan Malaysia, UKM Bangi, Selangor 43650, MY, Malaysia; b Graduate Business School, UCSI University, Kuala Lumpur, Malaysia

**Keywords:** Trust climate, Work engagement, Job performance, Turnover intention, China, Pakistan

## Abstract

While the significance of cross-cultural research has been acknowledged over time, there remains a gap in understanding its relationship with employee outcomes. This study delves into the associations between organizational trust climate (TC) and job performance (JP), as well as turnover intention (TI), seeking to ascertain if work engagement (WE) plays a mediating role. Additionally, the research investigates potential differences in the mediating effect between China and Pakistan. Data gathered from 270 participants in China and 242 in Pakistan were subjected to structural equation modeling (SEM) for analysis. Findings indicated that perceptions of individual WE serve as a bridge between organizational TC and JP, with the effect on JP being notably stronger among the Chinese participants. Moreover, the mediating role of WE in the link between organizational TC and TI was more pronounced for the Pakistani participants. On a practical front, such insights can equip managers with a nuanced understanding of the ripple effect that a trust-infused environment can have on employee engagement, subsequently influencing performance and retention rates.

## 1 Introduction

In today's global economy, organizational researchers focused on human resource management (HRM) often grapple with securing a competitive advantage amidst the rapidly evolving business landscape [1]. To navigate these challenges, organizations are compelled to broaden their operations and enhance performance, necessitating an introspective look into their employee dynamics. Ultimately, the success or failure of an organization largely hinges on its workforce [2,3]. It is a widely held belief that an organization's most exceptional employees constitute its competitive advantage [4]. The aim for organizations is to attract and retain these top talents while avoiding harmful and costly organizational consequences [5]. This calls for a committed and engaged workforce that not only aligns with the organizational values and objectives but also operates at peak potential [6,7]. Casteleiro Mendes [8] posited that in the modern competitive milieu, employee engagement has emerged as a pivotal determinant of organizational success. Hence, the inference is unequivocal: cohesive teams excel when founded on mutual trust.

Work engagement (WE) is defined as “a positive, fulfilling, work-related state of mind characterized by vigor, dedication, and absorption” [9]. *Vigor* entails high levels of energy and mental resilience during work. *Dedication* denotes a profound commitment to one's job, imbuing it with significance, inspiration, enthusiasm, challenge, and pride. *Absorption*, on the other hand, is typified by complete concentration and joyful immersion in one's tasks [9]. An engaged workforce is seen as crucial for sustaining a competitive advantage [6]. Given its association with employee performance and well-being, WE have garnered significant attention within many organizations. For instance, employee WE associate with numerous positive work outcomes, such as task performance [10], proactive coping strategies [11], innovation [12], citizenship behaviors [13], and creativity [14]. Hence, it is hardly surprising that HR professionals emphasize that maintaining an engaged workforce is paramount for organizations, necessitating consistent efforts [15]. Despite the noted benefits of WE, limited research explores the underlying drivers of workplace engagement.

Antecedents associated with WE encompass credible leadership [16], artificial intelligence [17], need for achievement [18], authentic leadership [19], organizational justice [20], fringe benefits [21], workaholism [22], and organizational tenure [23]. From this vantage, extant literature has evidenced that job resources (such as job control, supervisory coaching, social support, and performance feedback) and personal resources (e.g., optimism and self-efficacy) are pivotal in driving WE due to their inherent motivational properties [24]. Consequently, there is imperative to investigate a broader array of WE determinants to deepen the understanding of this construct [6]. While numerous studies have explored the nexus between trust and motivational outcomes organizational citizenship behavior, job satisfaction, and turnover intentions [25], the relationship between trust and WE remains relatively under-researched. Such observations indicate a potential linkage between trust climate (TC) and WE that remains to be elucidated.

Moreover, from the lens of HRM, there is a pressing need to delve deeper into the role of WE within workplaces. By gaining a nuanced understanding of the antecedents of WE and its linkage to employee behaviors, managers can better oversee the evolution of WE, positioning themselves to amplify its positive ramifications on employees. Employing the social exchange theory [SET; 26] as an anchoring framework, it is posited that a TC can bolster employees' sense of engagement at work, culminating in desirable employee outcomes such as enhanced job performance (JP) and diminished turnover intention (TI).

The primary goal of this study is to discern and elucidate whether WE can amplify the mechanisms underpinning a trusted climate, subsequently mitigating employee TIs and elevating their performance metrics, particularly within the contexts of China and Pakistan. In essence, this inquiry seeks to make two seminal contributions to the domains of TC and WE. *Firstly*, this empirical exploration evaluates: (a) the influence of TC on WE, JP, and TI; (b) the effect of WE on JP and TI; and (c) the mediating role of WE in the aforestated relationships. *Secondly*, by juxtaposing data from Chinese and Pakistani participants, the study discerns cultural variations in the mediation effects of employee WE on the dynamics between TC, JP, and TI. Specifically, this marks a pioneering effort in probing the impact of trust climate on employee job performance via work engagement in a cross-cultural milieu. While both cultures predominantly lean towards collectivism, inherent distinctions do prevail.

## 2 Theoretical foundation and hypotheses development

### 2.1 TC and WE

Over the past three decades, trust has emerged as a central theme in organizational research [27]. Trust is defined as “a psychological state encompassing the intention to accept vulnerability based upon optimistic anticipations of another's intentions or behavior” [28]. Extensive literature underscore trust paramount importance in forging robust human connections and fostering social relations [29], as well as its pivotal role in crafting a secure environment [30]. Previous research has predominantly scrutinized the ramifications of trust on social, political, and economic paradigms [31], suggesting that elevated levels of trust correlate with economic advancement [32], and economic autonomy [[33], [34]]. 34Moreover, trust stands out as a formidable determinant of myriad employment outcomes like job satisfaction and commitment [35], and JP [36]. Existing literature elucidates how trust facilitates collaboration, fortifies interpersonal bonds, and steers pivotal employee attitudes and behaviors [37]. Inherently, a trust-infused environment alleviates the imperative for surveillance, enabling individuals to immerse fully in their roles without apprehensions about potential malfeasance by peers [38]. Consequently, a logical deduction posits a connection between TC and employee WE.

The relationship between TC and employee WE is elucidated using SET as the theoretical underpinning. SET underscores “the significance of reciprocal exchange spurred by an organization's treatment of its members, anticipating that such gestures will be reciprocated in due course” [39]. Fundamentally, trust engenders a sense of obligation in employees to reciprocate the trust vested in them. This reciprocation often manifests as positive work attitudes, particularly heightened engagement. Relying on SET tenets, it is posited that elevated levels of TC are likely to associate positively with the “norms of reciprocity” inherent in an employee's sense of WE. Moreover, considering the foundational conceptual analysis of trust and WE [40], future studies should endeavor to explore the nuances of trust across diverse research contexts. The ensuing hypothesis encapsulates the above discourse.

H1 TC will be positively related to WE.

### 2.2 TC, JP, and TI

Limited research within the management domain illuminates the influence of TC on work outcomes such as JP and TI [41]. Dirks Ferrin [35] meta-analysis revealed a marginal positive association between trust in leadership and JP, yet the organizational climate of trust towards employees appears not to significantly influence JP. Further, certain researchers investigating trust in upper management vis-à-vis JP illustrated that trust could shape resource allocation between trustor and trustee [42]. Recently, Guinot Chiva [43] systematic review highlighted trust role in enhancing employee decision-making and JP. While numerous investigations underscore a positive link between trust and performance [42,44], contrasting findings from Mayer Gavin [42] meta-analysis, among others, report no such relationships [45,46]. Hence, existing insights into the TC-JP relationship are inconclusive.

Turning to TI as an outcome of TC, Dirks Ferrin [35] suggest that employees lacking trust-based relationships might exhibit detrimental tendencies, such as increased TI. Several studies across diverse contexts corroborate the nexus between trust and the inclination to exit an organization [6,47,48]. It seems that organizational trust fosters motivational and decision-making mechanisms, engendering feelings of support, achievement, and organizational loyalty [49]. Drawing from SET, trust presence in a relationship fosters positive, cooperative engagements, like enhanced JP [e.g., JP; 50], primarily as trust engenders a willingness to share resources without apprehension of deceit. Conversely, the absence of trust in relationships, especially in informal social exchanges that lack explicit future commitments, impedes growth. Based on these considerations, we propose the following.

H2 TC will be (a) positively related to JP and (b) negatively related to TI.

### 2.3 WE, JP, and TI

WE and its outcomes have garnered significant attention in academic literature. It has been generally observed that WE enhance employees’ JP [6] and mitigates TI [10]. Research on full-time dyad workers in the Singapore service sector has found that WE instigates in-role JP [51]. Rafiq et al. [6] carried out a study on healthcare workers in Pakistan, revealing that WE act as an antecedent to TI behavior.

Furthermore, the SET offers a theoretical lens to understand the relationship between employee WE, JP, and TI [e.g., 52]. SET posits that obligations emerge from the ongoing interactions between parties, rooted in mutual dependency [52]. When both employers and employees adhere to these exchange norms, employees tend to exhibit high energy, commitment, and concentration in their tasks. Such adherence fosters mutual trust, facilitating harmonious employer-employee relationships. As a byproduct, employees tend to exhibit enhanced performance, subsequently reducing detrimental outcomes, such as TI. Given these discussions, it becomes evident that the nexus between employee WE and its implications, like JP and TI, warrants further research [e.g., JP and TI; 53]. Therefore, in light of the aforementioned discourse, we postulate.

H3 The employee WE will be (a) positively related to JP and (b) negatively related to TI.

### 2.4 The mediating role of WE across culture

Having delineated the relationships above, emphasizing the mediating role of WE, we hypothesize that given TC predictive capacity for WE and related outcomes and given WE influence on these outcomes, WE serve as a conduit for the impact of TC on JP and TI. This mediating role differs when considering Chinese and Pakistani participants. While the centrality of trust in JP [36] and TI [6] is well-established, the intermediary role of WE has not been adequately explored in research. Furthermore, personal resources have been identified to mediate the relationship between challenging working conditions and job outcomes [54]. In particular, studies have validated that WE bridge the link between organizational factors, like social exchange relationships, and positive work outcomes. Rayton et al. [55] found that WE play a mediating role between alignment perspectives and work outcomes. Consequently, we argue that WE stand as a mediator between organizational TC and WE.

Concurrently, we posit that the mediating role of WE vary across the two collectivist cultures. Organizational culture is deemed pivotal and intriguing, given its influence on a myriad of organizational outcomes [56]. One of the most extensively studied dimensions in cultural values is the dichotomy between individualism and collectivism [57]. Ever since Hofstede's landmark work in the 1980s, collectivism has garnered broad consensus, spotlighting the degree to which individuals are integrated into in-groups [58]. These in-groups may encompass communities, families, or other social entities.

Although a plethora of studies on WE within Western cultures have been conducted, scant research specifically delves into the WE associated with Asian collectivistic cultures. The existing body of literature underscores the influence of collectivism on organizational practices and behaviors. For instance, Ramamoorthy Carroll [59] examined how collectivism impacts HRM practices, including recruitment, compensation, performance evaluations, workplace safety, training, and career development.

In light of the globalizing economy, scholars delving into HRM behaviors are pivoting towards cross-cultural research endeavors [60]. Among Asian economies, China notably eclipses others, including Pakistan, primarily attributed to its robust employment policies. Notably, Rafiq Weiwei [27] have highlighted that emerging economies, especially nations like Pakistan and China, are in the throes of profound structural and institutional metamorphosis. This raises an intriguing query: Despite their shared collectivistic cultural underpinnings, are Chinese employees inherently more engaged at work than their Pakistani counterparts? The objective of this study is to shed light on this poser. To the best of our understanding, no prior research has juxtaposed these collectivistic cultures, perhaps stemming from the presumption that cultural disparities within the domain of collectivism are marginal.

Disparities might be discernible between the Chinese and Pakistani cohorts concerning the mediating role of WE on the relationships between TC and JP, and between TC and TI, although extant research has not ventured into a comparative analysis of this dynamic. Wang Liu [61] suggest that current academic discourse provides ambivalent findings regarding the nexus between cultural orientations and their influence on employee engagement behaviors. Some scholars contend that employees from collectivist cultures might exhibit lower levels of workplace engagement as compared to those from individualistic orientations. Conversely, empirical studies have revealed that individuals from collectivist societies often possess enriched resources, including robust support systems from colleagues and kin, which augments their productivity and level of engagement within organizations [62]. Nevertheless, voices like Costigan et al. [63] and Cropanzano Mitchell [64] propound that, while reciprocity in social exchanges is a ubiquitous norm, its manifestation might differ when juxtaposing collectivist cultures with individualistic ones, suggesting it is not a one-size-fits-all phenomenon. This incongruence in academic insights underscores the imperative for further exploration of the interplay between collectivist cultures and engagement dynamics. Recognizing the drivers that amplify workplace engagement is pivotal. Thus, accordingly, we postulate that.

H4 The mediation effect of employee WE on (a) JP and (b) TI will be different for China participants and Pakistan participants.

## 3 Methodology

### 3.1 Sample and procedures

Data for this research was collated from full-time employees of two Chinese and three Pakistani media organizations. While the specific identities of these organizations are withheld to maintain anonymity, our sampling methodology was a simple random approach for both nations. The Chinese media organizations, located in Beijing, were approached with assistance from the Pakistan embassy in Beijing, utilizing their local contacts. Initially, an email detailing the study's objectives and institutional consent was sent to the senior management of the TV channel. Following a positive response, a formal meeting was arranged to administer our survey, with the Human Resource departments of the concerned organizations being apprised in advance. The HR directors of the participating organizations not only facilitated the survey by distributing the questionnaires but also advocated for participation, reinforcing that participation was voluntary and responses would be managed confidentially.

In Pakistan, we based our data collection criteria on Gallup Pakistan TV rating analysis and initially shortlisted the top fourteen media channels. Engaging with the top management and HR professionals of these channels, three out of the fourteen expressed interests in participating in our survey. The HR directors of these organizations were proactive in promoting participation and facilitating the distribution of our questionnaires. The measures employed in the China sample were replicated for the Pakistan sample. For China, out of the 290 questionnaires disseminated, 270 were returned, yielding a response rate of 93.10%. Conversely, in Pakistan, out of the 270 questionnaires shared, 242 were retrieved, marking an 89.60% response rate.

Final, data was collated from 270 Chinese and 242 Pakistani employees (N = 512). This collective sample comprised 60.5% males, 43.0% with master's degrees, 41.4% having 1–5 years of professional experience, and 42.8% aged between 26 and 35. Notably, 68.9% of the respondents were married. The sample size for both nations—China (N = 270) and Pakistan (N = 242)—surpassed the recommended sample size to discern medium effects.

### 3.2 Measures

All scale items were anchored in prior research. Specifically for the China sample, the translation and back-translation technique [65] was employed to mitigate potential semantic inconsistencies between English and Chinese versions. We utilized a five-point Likert-type scale for all study constructs, where 1 equated to “strongly disagree” and 5 to “strongly agree".

*TC*: TC was measured using a 14-item scale, adapted from the scale originally developed and tested by Donovan et al. [66]. A sample item includes “Employee's complaints are deal with effectively”. The respective coefficient alphas were 0.94 (for China and 0.97 for Pakistan.

*WE*: We adopted WE with a 9-item version of the Utrecht WE Scale (UWES) scale from Ref. [67]. It includes three sub-dimensions: vigor, dedication, and absorption, each represented by three items. Examples include “At my work, I feel bursting with energy”, “My job inspires me”, and “I get carried away when I am working”. The respective coefficient alphas were 0.92 for China and 0.95 for Pakistan.

*JP*: We adopted JP with a 7-item scale from Williams Anderson [68]. Respondents indicated their engagement in behaviors such as “Engages in activities that will directly affect his/her performance evaluation.” The respective coefficient alphas were 0.91 for China and 0.94 for Pakistan.

*TI*: We evaluated TI with a 5-item measure introduced by Crossley et al. [69]. Previous job embeddedness s research employed the same owing to its design which mitigates overlap between job search and job attitude measurements [70]. The sample item is “I will quit this organization as soon as possible”. The respective coefficient alphas were 0.86 for China and 0.90 for Pakistan.

*Control variables*: For this study, various demographics were included as control variables, recognizing their potential association with our study constructs. We thus controlled factors such as gender, marital status, education, and organizational tenure.

### 3.3 Data analysis

**Fig. 1:**
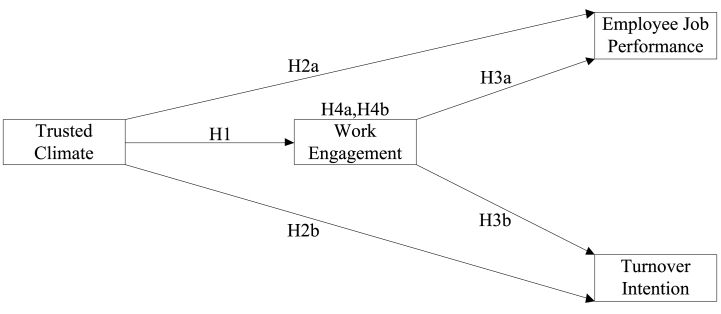
A hypothesized model.

First, a confirmatory factor analysis (CFA) was conducted using the AMOS 24 package for both nations to validate the discriminant and convergent validity of our four identified constructs: WE, TC, TI, and JP. Subsequently, to probe mediation, we applied structural equation modeling (SEM) complemented by model fit indices analysis. We further employed bootstrapping techniques to validate the mediational indirect effects [71]. Mediation is theoretically present when the path coefficients ‘a’ (from TC to WE) and ‘b’ (from WE to employee JP) are statistically significant, and the 95% confidence interval (CI) for the standard error (SE) of the standardized indirect effects (a × b) does not encompass zero [71,72]. Finally, to discern the moderated mediation effect, we examined the equality of a specific parameter ‘b' (pertaining to the intervening-outcomes variable) within each posited model. The hypothesized mediated model is presented in Fig. 1.

## 4 Results

### 4.1 Preliminary analysis

Table 1 provides the inter-correlations, summary statistics, and psychometric data for the variables in the China and Pakistan samples. As depicted in Table 1, all hypothesized relationships of the study align with expectations. The Variance Inflation Factor (VIF) outcomes indicate acceptable multicollinearity levels, with VIF value at 1.82 [73].

**Table 1:** Variable inter-correlations, summary statistics, and psychometric properties.

Variable	*China* sample		*Pakistan* sample			2	3	4	5	6	7	8
	M	SD	M	SD	1							
1. Turnover intention	1.86	.73	2.09	.82	–	−.41**	−.46**	−.47**	.09	−.11	−.10	−.14*
2. Job performance	4.30	.67	4.20	.86	−.65**	–	.70**	.68**	−.04	.07	.04	−.06
3. Work engagement	4.24	.65	4.16	.80	−.43**	.53**	–	.61**	−.02	.09	.06	.02
4. Trust climate	4.31	.61	4.19	.86	.35**	.53**	.54**	–	−.05	.05	.10	−.01
5. Gender	.61	.48	.60	.49	−.02	.08	.07	.08	–	−.08	.04	−.09
6. Marital status	.72	.45	.66	.47	−.04	.01	.10	.01	.03	–	.18**	.57**
7. Education	2.51	.88	2.50	.91	−.01	.04	.06	.03	.02	.26**	–	.26**
8. Organizational tenure	2.94	1.48	2.91	1.38	.14*	−.11	−.03	−.04	−.02	.23**	.27**	–

**Note:** M = mean; SD = standard deviation; figures above the diagonal are for the China sample; those below the diagonal are for the Pakistan sample.

*p < 0.05.

**p < 0.01.

### 4.2 Confirmatory factor analysis

Before examining our hypotheses, we employed confirmatory factor analyses to: (1) verify the distinctness of our research constructs (namely, 10.13039/100006922TC, WE, employee JP, and 10.13039/100004361TI) for each national sample; and (2) support the equivalence of the constructs in both China and Pakistan. For gauging the measurement of the hypothesized model, we employed the absolute fit index to contrast our four-factor model with various alternative models, irrespective of their data fit [74]. We adopted the subsequent criteria for evaluating the fit indices [75,76]: 1) Comparative Fit Index (*CFI* > 0.90); 2) Tucker-Lewis Index (*TLI* > 0.90); and Root-Mean-Square Error of Approximation (*RMSEA* < 0.05). Results revealed that the anticipated four-factor measurement model fit the data better using both the China dataset (*χ*^2^_554_ = 840.771; CFI = 0.95; TLI = 0.95; RMSEA = 0.04) and the Pakistan dataset (*χ*^2^_554_ = 1004.362; CFI = 0.94; TLI = 0.93; RMSEA = 0.05) in comparison to potential alternative models. Both datasets adequately substantiated the discriminant validity, suggesting that our study is not affected by self-reported measures.

Delving deeper into the cross-cultural construct validity, Tsui et al. [77] posited that merely relying on translation/back-translation is not sufficient. To ascertain if the factor structure was uniform across respondents from China and Pakistan, we juxtaposed a four-factor model—with factor correlations, loadings, and error variances held constant across the two datasets—to a less restrictive model where factor loadings were freely estimated for both countries [see 78]. The results manifested robust support for both the constrained model (*χ*^2^_1110_ = 1940.415; 10.13039/100008176CFI = 0.94; RMSEA = 0.03) and the less restrictive model (*χ*^2^_1146_ = 1968.840; 10.13039/100008176CFI = 0.94; RMSEA = 0.03). Additionally, the chi-square difference between these models was statistically insignificant (Δ*χ*^2^_36_ = 28.425; *n. s*). Hence, the evidence sufficiently justified employing identical measures concurrently for both countries, underscoring that response bias is unlikely to pose a concern for China and Pakistan.

### 4.3 Hypotheses testing

**Fig. 2:**
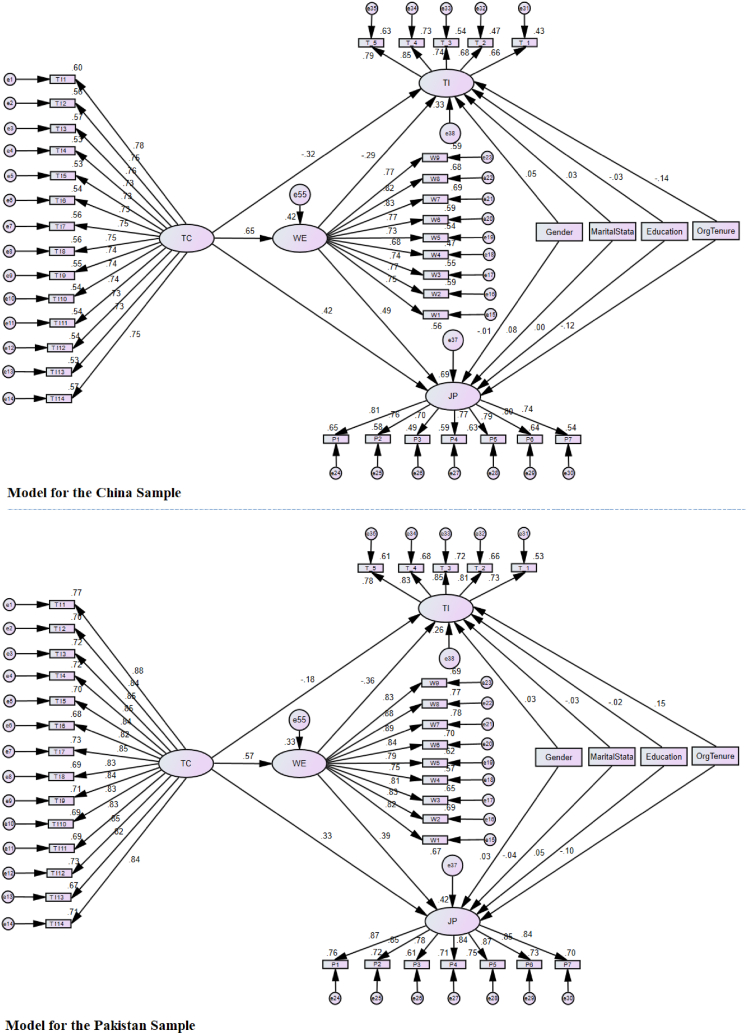
A cross-cultural comparison of mediating model of WE in the relationship between TC – employee JP and TI relationships (***p < 0.001; *p < 0.05).

The hypothesized research model is presented in Fig. 1. Results reported in Fig. 2 illustrated that Hypothesis 1 was supported for both countries, with 10.13039/100006922TC having a substantial positive effect on WE (*β*_*China*_ = 0.65 and *β*_*Pakistan*_ = 0.57). A review of the results clearly indicates that the empirical evidence supports Hypotheses 2a and 2 b Specifically, 10.13039/100006922TC exhibits a positive association with JP (*β*_*China*_ = 0.42 and *β*_*Pakistan*_ = 0.33) and an inverse relation with 10.13039/100004361TI (*β*_*China*_ = −0.32 and *β*_*Pakistan*_ = −0.18). When evaluating hypotheses 3a and 3 b, the results in Fig. 2 show that WE is positively associated with JP (*β*_*China*_ = 0.49 and *β*_*Pakistan*_ = 0.39) and negatively with the TI (*β*_*China*_ = −0.29 and *β*_*Pakistan*_ = −0.37). Consequently, hypotheses 3a and 3 b are confirmed.

Additionally, hypotheses 4a and 4 b delve into the mediating role of WE in the relationships between organizational TC-JP and TI, noting variations between countries. To assess this, a moderated mediation analysis was executed. First, for Hypothesis 4a, *China* sample results show that the model has a statistically significant mediation effect. For Hypothesis 4a, results from the China sample indicate a statistically significant mediation effect: indirect effect (a × b = 0.30) with bounds ranging from 0.212 to 0.407, both of which exclude zero. Similarly, the *Pakistani* sample exhibits a significant mediation effect: indirect effect (a × b = 0.119) with bounds between 0.131 and 0.273, which also exclude zero. For Hypothesis 4 b, the *China* sample indirect effect was (a × b = −0.225) with bounds of −0.384 to −0.110, and *Pakistan* was (a × b = −0.069) with bounds of −0.123 to −0.025. In both cases, zero was excluded, confirming the statistical significance of the mediation effect for both countries.

Subsequently, a multi-group comparison was conducted. Following the guidelines set by followed MacKinnon [79], we examined the moderated mediation effect by comparing the path coefficients ‘b’ (WE – JP and TI) within each mediation model across groups.

Results showed that the path coefficients for WE – JP (*β*_*China*_ = 0.49 and *β*_*Pakistan*_ = 0.39) and for WE – TI (*β*_*China*_ = −0.29 and *β*_*Pakistan*_ = −0.36) significantly differed based on their critical ratios (Critical Ratio >1.96). This distinction highlights the statistical differences between China and Pakistan concerning how WE mediates the relationships between TC – JP, and TI. Further, the mediation effect posited in Hypothesis 4a is more pronounced for Chinese participants, while the effect described in Hypothesis 4 b is stronger among Pakistani participants. As a result, both hypothesis 4a and 4 b receive empirical support (refer to Fig. 2).

## 5 Discussion and conclusion

This study aimed to delve into the direct relationships between employees' TC and WE, TC-JP and TI, as well as WE-JP and TI. Additionally, the research examined the mediation effect of WE within the TC – JP and TI relationships. The findings derived from the SEM largely validated all hypothesized association. Significantly, the mediating influence of WE in the TC – employee JP relationship was more prominent for participants from China, while the mediation impact of WE in the TC – TI association was more pronounced among participants from Pakistan. The key insights are discussed below in relation to both countries.

### 5.1 Empirical and theoretical contribution

Consistent with prior studies, one of the core findings pertains to the strong and positive relationship between TC and WE (hypothesis 1) in both Chinese and Pakistani organizations. The positive association between TC and WE highlighted in our results reinforces the importance of an organization's trustworthiness in driving employee engagement. Notably, our study delineates the varying magnitudes of this association between the two countries, with Chinese employees demonstrating a more pronounced engagement response to perceived TC than their Pakistani counterparts. This heightened response in the Chinese context could be attributed to cultural and organizational values that emphasize trust and harmony as central to workplace relationships, as elucidated in earlier research [27]. Conversely, the slightly subdued relationship in Pakistan could be influenced by other unexplored organizational or societal factors, suggesting a need for a more context-specific investigation in future studies [57].

Expanding upon the seminal work of Donovan et al. [66], our investigation confirmed direct associations between TC and both JP and TI (as posited in hypothesis 2). A strong and positive organizational TC predisposes employees to exhibit heightened JP and reduced tendencies to contemplate leaving. Notably, our findings reveal subtle differences in the dynamics of these associations across the two examined nations. Specifically, the bond between TC and JP appeared to be more robust in the Chinese context than in Pakistan. This could be anchored in the deep-seated Chinese cultural values that emphasize diligence and commitment in response to trust, as elucidated in previous studies [27]. In contrast, the relationship between TC and TI was more pronounced in Pakistan, suggesting that a trust-laden organizational atmosphere in Pakistan might play a more significant role in employees' stay-or-leave decisions, potentially due to unique socio-cultural or organizational dynamics specific to the region [57].

In the domain of WE and its interplay with JP and turnover intention (TI) — central to hypothesis 3 — our findings divulge intriguing variances when juxtaposing the Chinese and Pakistani corporate milieu. As illuminated by past studies [10], elevated WE invariably augments JP and concurrently attenuates TI. Yet, the intensity of these associations differs noticeably between China and Pakistan. Drawing upon cultural motivation theories, the heightened tie between WE and JP in China may resonate with the nation's cultural orientation towards achievement and diligence [56,61]. On the other flank, the pronounced inverse linkage between WE and TI in Pakistan can be contextualized against the nation's political and collectivist values, where high engagement might engender a stronger sense of loyalty and community belonging, dissuading thoughts of departure [57]. This dichotomy not only amplifies the intricate interplay of culture and engagement but also echoes sentiments encapsulated in prior cross-cultural research [56,57].

In addressing Hypothesis 4a, we discerned that the mediating influence of WE between TC and JP was more pronounced for Chinese participants. Both East Asia and South Asia typify “acknowledge us” regions emblematic of collectivist cultures [80]. In such settings, trust and engagement are frequently viewed as synonymous when assessing employees’, WE [6]. However, the manner in which engagement is cultivated varies based on cultural nuances [81]. Notably, while the manner and intensity of engagement effects diverge by nation, such distinctions exist even within the same cultural cluster, as seen between China and Pakistan [e.g., China and Pakistan; 80]. Positive psychological climates, such as TC, offer a competitive edge in the Chinese context, spurring employees to commit more profoundly, and contribute their personal resources to their organizations, culminating in enhanced task performance [7]. Yet, our exploration yielded scant prior research in Pakistan that corroborates these observations, although the evidence distinctly favors our hypothesis for China.

For Hypothesis 4 b, the mediating effect of WE between TC and TI emerged stronger for Pakistani participants. This aligns with Costigan et al. [82] findings, emphasizing that the association between TC and turnover intentions exhibits variability across countries. Pakistan's results can be attributed to two pivotal reasons. Firstly, Pakistan's relatively subdued collectivism score [80], in contrast to China's, might precipitate heightened TI. More collectivist societies often witness members expending greater effort at work, perceived as making sacrifices for their in-group, and drawing from robust work support systems. Conversely, Pakistan grapples with economic challenges more acute than many of its Asian counterparts [83,84], a milieu wherein employees often grapple with extended work hours, stagnant salaries, and more. Consequently, their engagement remains superficial, with waning trust that eventually fosters pronounced TI.

From a theoretical standpoint, this study enriches the SET landscape by unraveling the intricate dynamics of TC, WE, JP, and TI. Traditional SET postulates that relationships evolve based on mutual exchanges where reciprocated benefits are expected. By highlighting the nuanced interplay between TC and employee outcomes in diverse cultural contexts, we underscore the significance of creating symbiotic organizational relationships. Our findings demonstrate that trust, as an underpinning factor of the exchange process, intensifies the quality of the exchange, thus amplifying work engagement and shaping consequential job behaviors. This novel application and contextual extension of SET offers scholars a refined lens to scrutinize organizational relationships in multicultural settings.

### 5.2 Practical implications

From a pragmatic perspective, this study offers several salient insights. Our results underscore the importance for employers, regardless of their cultural milieu, to recognize the significance of fostering a trustful climate and enhancing employee engagement. This becomes even more pertinent in collectivist cultures where the sanctity of committed relationships is paramount [85,86]. Collectivist societies display a heightened sensitivity to a trustworthy organizational ambiance. Once established, it augments employees' WE, which in turn positively influences their attitudes and behaviors. Given that individual WE is a paramount concern in today's competitive environment, our findings shed light on how individuals' perceptions of organizational TC boost their sense of enhanced WE. This, in the long run, motivates them to excel in their roles. As organizations increasingly adopt global cultural norms, discerning the elements to emphasize in hierarchical versus egalitarian cultures becomes a noteworthy addition to this discourse.

### 5.3 Limitations of study

This study presents several limitations warranting attention. First and foremost, the research narrows its focus to individuals employed within the media industry. Such individuals might exhibit job attitudes distinct from their counterparts in alternative sectors [87]. To augment the generalizability of our findings, future inquiries should span a broader array of organizational settings. Secondly, our research encompassed both collectivistic and individualistic cultures. While both are typically categorized as collectivist, discernible nuances exist. Subsequent studies might delve into leveraging culture as a potential moderating variable [88]. Lastly, the sole reliance on self-reported measures presents a potential pitfall. The marked significance in correlations suggests a possible mono-method bias across the evaluated variables. Utilizing a solitary source might induce biases of a common method [89]. Future scholarship should be focused on multiple sources within organizations to reduce such bias.

## 6 Conclusion

This research explored the intricate dynamics between TC, WE, JP, and TI within Chinese and Pakistani organizational contexts. Our findings underscore the pivotal role of TC in enhancing WE, with the subsequent positive impact on JP and reduction in TI. Moreover, regional nuances emerged, revealing stronger associations in China between TC and JP, while Pakistan showed pronounced relationships between TC and TI. These outcomes not only reaffirm the universality of trust in influencing employee attitudes but also emphasize the need for organizations to appreciate cultural differences in operationalizing trust. By understanding these interactions, businesses can tailor their strategies to foster both employee engagement and organizational success, irrespective of their cultural milieu.

## Funding statement

This work is supported by 10.13039/501100004515Universiti Kebangsaan Malaysia, Malaysia; under grant number EP-2020-106.

## Author contribution statement

Aini Aman: Conceived and designed the experiments; Performed the experiments; Analyzed and interpreted the data; Contributed reagents, materials, analysis tools or data; Wrote the paper.

Muhammad Rafiq: Conceived and designed the experiments; Performed the experiments; Analyzed and interpreted the data; Contributed reagents, materials, analysis tools or data; Wrote the paper.

Omkar Dastane: Conceived and designed the experiments; Analyzed and interpreted the data; Wrote the paper.

## Data availability statement

Data will be made available on request.

## Declaration of competing interest

The authors declare that they have no known competing financial interests or personal relationships that could have appeared to influence the work reported in this paper
